# Bacillary layer detachment in acute Vogt-Koyanagi-Harada disease: an early predictor of long-term complications in a Brazilian cohort

**DOI:** 10.1186/s40942-025-00673-6

**Published:** 2025-04-22

**Authors:** Ruy Felippe Brito Gonçalves Missaka, Mauro Goldbaum, Cleide Guimarães Machado, Emmett T. Cunningham, Fernanda Maria Silveira Souto, Marcelo Mendes Lavezzo, Priscilla Figueiredo Campos da Nóbrega, Camillo Carneiro Gusmão, Viviane Mayumi Sakata, Maria Kiyoko Oyamada, Carlos Eduardo Hirata, Joyce Hisae Yamamoto

**Affiliations:** 1 Department of Ophthalmology, LIM-33, Faculdade de Medicina FMUSP, Universidade de Sao Paulo, Sao Paulo, 05402-000 SP Brazil; 2 West Coast Medical Group, San Francisco, CA USA; 3 Department of Ophthalmology, California Pacifical Medical Center, San Francisco, CA USA; 4 Department of Ophthalmology, Stanford University School of Medicine, Stanford, CA USA; 5 The Francis I Proctor Foundation, UCSF School of Medicine, San Francisco, CA USA

**Keywords:** Biomarkers, Inflammation, Optical coherence tomography, Uveitis, Uveomeningoencephalitic syndrome

## Abstract

**Background:**

Long-term complications occur in some patients with Vogt-Koyanagi-Harada disease (VKHD).This study aimed to evaluate the presence of bacillary layer detachment (BALAD) at presentation as an early predictor of long-term structural and functional outcomes in a Brazilian cohort of patients with VKHD.

**Methods:**

Retrospective, clinic-based cohort study in Sao Paulo, Brazil, including 33 patients (66 eyes) with acute VKHD systematically followed for 12 months, since presentation. Clinical and multimodal data collected on spectral domain optical coherence tomography (SD-OCT) were analyzed at baseline and months (M) 1, 3, 6, 9 and 12. Correlations between OCT-based structural findings and the occurrence of subretinal fibrosis (SRFib), sunset glow fundus (SGF), and nummular chorioretinal lesions (NCL) at month 12 were studied. Outcomes were presence of retinal changes on SD-OCT during the study period; risk factors for SRFib, SGF and NCL at month 12. Univariate, bivariate, and multivariate analyses were employed.

**Results:**

At baseline, serous retinal detachments (SRD) were observed in 96.4% and BALAD in 48.2% of 56 eyes; at M1, SRD were observed in 42.4% and BALAD in 6.1% of 66 eyes. Subretinal fluid (SRFld) was still present in 9.1% at M3, in 4.5% at M6 and in 3.0% of eyes at M9. Using bivariate analysis, the early presence of BALAD was associated with a larger SRD area (*p* = 0.036) at presentation, and with the presence of both SRFib (*p* < 0.001) and SGF (*p* = 0.008) at M12. Using multivariate regression analysis, both early BALAD (OR, 12.04; *p* = 0.011) and a longer interval to treatment start (32 vs. 17 days; OR, 1.17; *p* = 0.004) were each independently associated with the formation of SRFib at M12, whereas both early BALAD (OR, 14.78; *p* = 0.002) and anterior uveitis recurrences (OR, 30.94; *p* = 0.022) were each associated with the development of SGF. The late occurrence of NCL was not associated with the presence of BALAD at presentation.

**Conclusions:**

In acute VKHD, the presence of BALAD at presentation was associated with a markedly increased long-term risk of developing both SRFib and SGF.

**Supplementary Information:**

The online version contains supplementary material available at 10.1186/s40942-025-00673-6.

## Background

Vogt-Koyanagi-Harada disease (VKHD) is a systemic autoimmune disease that affects tissues rich in melanocyte-derived proteins, such as the choroid, inner ear, meninges, and skin. Vogt-Koyanagi-Harada disease is an important cause of noninfectious uveitis, mainly in Asians, Middle Easterners, Latinos and Native Americans [1]. In Brazil, it is the most common noninfectious uveitis in tertiary care facilities [2]. An early diagnosis followed by prompt and adequate therapy can result in complete recovery of vision with no lasting sequelae. However, some patients develop permanent long-term complications, such as subretinal fibrosis (SRFib), sunset glow fundus (SGF) and nummular chorioretinal lesions (NCL) or scars, despite appropriate therapy [3, 4]. We therefore sought to investigate predictors of long-term complications using multimodal imaging-based findings of early disease severity biomarkers in VKHD [5–7]. 

Septations seen on optical coherence tomography (OCT) have been proposed as part of a set of classification criteria for acute VKHD [8]. Mehta et al. defined these septations producing a splitting at the myoid zone as “bacillary layer detachments” (BALAD) [9]. Such BALADs have been observed in a variety of inflammatory and non-inflammatory diseases [10]. According to Agarwal et al., BALAD is particularly common in the acute phase of VKHD [11], but the precise prognostic importance of this finding in VKHD patients remains unknown. The purpose of this study was to report the frequency of BALAD in acute VKHD Brazilian patients and to investigate its association with structural and functional outcomes during the 12-month follow-up from disease onset.

## Methods

This is a retrospective, clinic-based cohort study of consecutive patients diagnosed with acute VKHD [12], attended in the Uveitis Service, Hospital das Clinicas HCFMUSP, Faculdade de Medicina, Universidade de Sao Paulo, Sao Paulo, Brazil, from June 2011 to March 2021. This study was approved by the Institutional Ethics Committee (CAAE: 29270620.7.0000.0068) and followed the tenets of the Declaration of Helsinki.

### Study subjects

Thirty-three patients with VKHD (66 eyes) were included. Data analyzed were best-corrected visual acuity (BCVA), anterior chamber cells, treatment, full-field electroretinogram (ffERG), and imaging evaluations at diagnosis (M0), 1st (M1), 3rd (M3), 6th (M6), 9th (M9) and 12th (M12) months (M). Treatment involved methylprednisolone pulse therapy followed by oral prednisone tapering associated or not with oral immunosuppressive therapy (IMT). Multimodal imaging comprised fundus photography, autofluorescence, fluorescein (FA) and indocyanine green (ICGA) angiographies, and spectral domain (SD) OCT. Patients with incomplete data and/or with other ocular disorders were excluded. The quantification of FA and ICGA images was based on the inflammatory score for uveitis published by the Angiography Scoring for Uveitis Working Group [13]. A subnormal full-field electroretinogram was defined as having at least one parameter below the 5th percentile of healthy age and gender-matched controls.

### Fundus imaging

Optical coherence tomography and enhanced depth imaging (EDI-) OCT were performed using spectral domain OCT (Spectralis^®^ HRA + OCT, Heidelberg Engineering, Germany). Nine millimeters of single horizontal and vertical line scans centered on the fovea, covering an area of 30° (minimum ART 25), were analyzed. For BALAD, an additional three-dimensional volume scans of the posterior pole of 25 horizontal line scans (minimum ART 25) were analyzed. The FA and ICGA (Spectralis^®^ HRA + OCT, Heidelberg Engineering, Germany) were analyzed at M0, M1 and M12.Color fundus photographs (Topcon TRC-50DX, Tokyo, Japan) at M12 were analyzed for the presence of SRFib, SGF and NCL [12, 14, 15]. 

### Analysis of optical coherence tomography

The anatomical landmarks and nomenclature of OCT structures adhered to the IN-OCT consensus [16] and the following parameters were analyzed: serous retinal detachment (SRD), bacillary layer detachment (BALAD), ellipsoid zone (EZ) and the interdigitation zone, retinal pigment epithelium (RPE) undulations and subfoveal choroidal thickness. The BALAD was defined as a “break within myoids that isolates ellipsoids and outer segments from the remainder of the cell bodies” [9]. The integrity of the ellipsoid and interdigitation zones were classified as intact, partially intact (when there was any disruption) or absent. The RPE undulations were classified into three grades, based on the appearance of undulations, as grade 1 (absent or slight), grade 2 (moderate) and grade 3 (severe) [7]. All imaging reading was carried out by three independent retina specialists (R.F.B.G.M, M.G. and C.G.M). In readings with complete disagreement, the image was collectively analyzed to achieve a final consensus.

In our sample of 66 eyes, not all images had adequate quality for analysis at baseline: SRD and BALAD were analyzed in 56 eyes, ellipsoid zone and interdigitation zone were analyzed in 55 eyes, and RPE undulations were analyzed in 53 eyes. Thereafter, all 66 eyes have images with minimal quality for analysis of all parameters, except at M12 there were 64 eyes for analysis, due to a patient (2 eyes) who missed the consultation.

Outcomes evaluated were the presence of retinal changes observed on SD-OCT during the study period and the risk factors for SRFib, SGF and NCL at M12.

### Statistical analysis

Statistical analysis was performed using SPSS software version 22.0 (SPSS Inc., Chicago, IL). Descriptive statistics included univariate means, medians, and ranges. Best-corrected visual acuity was converted to logMAR units. Bivariate associations were assessed using Spearman’s rho. For comparisons of continuous variables, the Mann-Whitney U test was applied for non-normally distributed data, while the Student’s t-test was used for normally distributed variables, as appropriate. Generalized estimation equations (GEE) with different link functions were employed for binary ocular data analysis. Multivariate logistic regression analyses utilized the Wald test. No corrections were applied for multiple testing. Time to resolution of OCT findings was compared across intervals using the median test, with intervals converted to numerical values and then interpreted back to their original time ranges.

## Results

All 33 patients (66 eyes) were followed from acute onset of the disease. Their mean (± SD) and median (interval) age was 33.6 ± 11 years and 33 years (range 15–67), respectively, at the time of diagnosis and 29 patients (87.9%) were female. The mean (± SD) and median (interval) of initial BCVA were 1.32 ± 0.83 (Snellen equivalent: 20/400) and 1.8 logMAR (Snellen equivalent: 20/1260) ranging 0 to 2.3, respectively; at M1, they were 0.39 ± 0.68 (Snellen equivalent: 20/50) and 0.2 logMAR (Snellen equivalent: 20/30) ranging 0 to 3, respectively; and at M12, they were 0.05 ± 0.14 (Snellen equivalent: 20/22) and 0 logMAR (Snellen equivalent: 20/20) ranging 0 to 0.7, respectively. Table 1 summarizes the socio-demographic and clinical data.

**Table 1:** Socio-demographic and clinical features of 33 patients (66 eyes) with Vogt-Koyanagi-Harada disease followed for 12 months since acute disease onset

Features		Presence of BALAD at baseline (*n* = 56 eyes)		*P*
		Yes (*n* = 27 eyes)	No (*n* = 29 eyes)	
Age at disease onset, years, mean ± SD	33.6 ± 11	32.07 ± 8.6	32.9 ± 7.6	0.756
median (interval)	33 (15–67)	35 (15–45)	32 (15–46)	
Male/Female, n (%)	4 (12) /29 (88)	2/15	2/16	
Interval to treatment start, days, mean ± SD	24.4 ± 16.0	21.48 ± 11.18	24.55 ± 15.19	0.483
median (range)	21 (3–67)	21 (8–48)	21 (3–51)	
Disease duration, months, mean ± SD	89.9 ± 25.9	84.2 ± 24.17	86.9 ± 23.25	0.721
median (interval)	85 (45–139)	83 (45–139)	82 (45–126)	
Visual acuity, logMAR, at M0 mean ± SD	1.3 ± 0.8	1.6 ± 0.8	1.09 ± 0.8	0.058
median (interval)	1.8 (0–2.3)	1.8 (0–2.3)	0.8 (0–2.3)	
at M1 mean ± SD	0.4 ± 0.7	0.32 ± 0.48	0.31 ± 0.56	0.532
median (interval)	0.2 (0–2)	0.2 (0–2)	0.1 (0–2)	
at M12 mean ± SD	0.05 ± 0.1	0.001 ± 0.008	0.0607 ± 0.16	0.214
median (interval)	0 (0–0.7)	0 (0–0.04)	0 (0–0.7)	
Fluorescein angiography total score at baseline				0.708
mean ± SD	6.7 ± 2.9	8.08 ± 2.03	6.04 ± 2.96	
median (interval)	8 (0–13)	9 (4–13)	7 (0–9)	
Full-field electroretinogram at M12, eyes (%) ^a^				
Subnormal	47 (75.8)	20 (74.0)	18 (62.0)	0.319
Treatment, patients (%)				0.823
Corticosteroid monotherapy	10 (30.0)	4 (12)	6 (18)	
Corticosteroid + early immunosuppressive therapy (≤ 1mo)	11 (34.0)	5 (16)	6 (18)	
Corticosteroid + late immunosuppressive therapy (> 1mo)	12 (36.0)	8 (24)	4 (12)	
Fundus outcomes in the first year of disease, eyes (%)				
Subretinal fibrosis	20 (30)	14 (51.9)	4 (13.8)	**< 0.001**
Sunset glow fundus	38 (57.5)	22 (81.5)	12 (41.4)	**0.008**
Atrophic nummular lesions	26 (39.3)	14 (51.9)	8 (27.6)	0.278

^a^ Sixty-two eyes were assessed at M12. Subnormal full-field electroretinogram was defined as having at least one parameter below the 5th percentile of healthy age and gender-matched controls

At baseline, on OCT, SRD was observed in 96.4% (54/56 eyes) and BALAD was detected in 48.2% (27/56 eyes) (Table 2); fluorescein pooling on FA were observed in 56.0% (37/66 eyes). The mean (± SD) and median (interval) subfoveal choroidal thickness at M1 were 465.95 ± 129.52 μm and 459 μm (range 218–784 μm), respectively.

**Table 2:** Spectral-domain optical coherence tomography findings in 66 eyes of 33 patients with Vogt-Koyanagi-Harada disease followed for 12 months since acute disease onset

OCT Findings, eyes (%)	Intervals					
	M0	M1	M3	M6	M9	M12
**Serous retinal detachment**	54 (96.4)	28 (42.4)	6 (9.1)	3 (4.5)	2 (3.0)	0 (0)
**Bacillary layer detachment**	27 (48.2)	4 (6.1)	0 (0)	0 (0)	0 (0)	0 (0)
**Ellipsoid zone integrity**						
Intact	0 (0)	6 (9.1)	16 (24.2)	24 (36.4)	29 (43.9)	31 (48.2)
Partially intact	41 (74.5)	55 (83.3)	49 (74.2)	42 (63.6)	37 (56.1)	33 (51.6)
Absent	14 (25.5)	5 (7.6)	1 (1.5)	0 (0)	0 (0)	0 (0)
**Interdigitation zone integrity**						
Intact	0 (0)	0 (0)	1 (1.5)	2 (3)	4 (6.1)	5 (7.8)
Partially intact	4 (7.3)	9 (13.6)	24 (36.4)	33 (50.0)	40 (60.6)	38 (59.4)
Absent	51 (92.7)	57 (86.4)	41 (62.3)	31 (47.0)	22 (33.3)	21 (32.8)
**Retinal pigment epithelium undulations**						
Absent or slight	21 (36.9)	57 (86.4)	66 (100)	66 (100)	66 (100)	64 (100)
Moderate	15 (28.3)	6 (9.1)	0 (0)	0 (0)	0 (0)	0 (0)
Severe	17 (32.1)	3 (4.5)	0 (0)	0 (0)	0 (0)	0 (0)

M: month

Regarding treatment, the mean (± SD) and median (interval) from symptoms onset to diagnosis/treatment were 24.45 ± 16.01 days and 21 days (range 3–67 days). After methylprednisolone pulse therapy, all patients were treated with 1 mg/kg/day prednisone with a slow taper for at least 6 months. Twenty-three patients (69.7%) received oral azathioprine (2 mg/kg/day); in 11 patients (34%), the immunosuppressant was added within first month of the disease. Azathioprine was replaced by mycophenolate mofetil in four refractory cases. At the end of the first year, 19 patients continued to use azathioprine and 4 patients were using mycophenolate mofetil. At M12, 22 patients had oral prednisone with a median dose of 10 mg/day (range 2.5-50 mg/day). Their mean (± SD) and median (interval) disease duration at inclusion was 89.9 ± 25.9 months and 85 months (range 45–139 months), respectively.

In the first year, SRFib was observed in 20 eyes (30%), SGF in 38 eyes (57.5%) and NCL in 26 eyes (39.3%). Subnormal ffERG was observed in 47 eyes (76.0%) at M12.

### Spectral-domain optical coherence tomography features

Serous retinal detachment was observed at baseline in 54 of 56 eyes (96.4%). In the first month, the subretinal fluid (SRFld) was still observed in 28 of 66 eyes (42.4%) and persisted to the 9th month in two eyes (3.0%). The median time to resolution of SRD was within 1 month, ranging from within the first month to 12 months. Early BALAD was observed in 27 of 56 eyes (48.2%) at baseline. One month after starting treatment, 4 of 66 eyes (6.1%) still had BALAD, and afterwards, no eyes showed the finding. The median time to resolution was within 1 month, ranging from within the first month to 3 months.

The ellipsoid zone, at M0, was partially intact in 41 of 55 eyes (74.5%) and absent in 14 eyes (25.5%); there was no eye with intact EZ. At M1, 6 eyes (9.1%) had completely recovered the EZ and, from the sixth month onwards, an intact or partially intact EZ was identified in all eyes. The median period for EZ recovery was between 3 and 6 months, ranging from within the first month to 12 months. It was observed that eyes with subnormal ffERG at 12 months had, in a greater proportion, absent or partially intact EZ in M3, 100% and 82.2%, respectively, when compared to eyes that evolved with normal ffERG, 0% and 17.8%, respectively, (*P* = 0.04). The interdigitation zone (IZ) was classified as absent in 51 of the 55 eyes (92.7%) at M0. Even after the third month, 41 eyes (62.3%) did not have this line. The median time for IZ recovery was 9 months, ranging from 1 to 12 months.

Undulations of RPE-Bruch membrane complex were graded as severe in 17 eyes (32.1%) at M0; at M1 only three eyes (4.5%) still had severe undulations. The characteristics observed in the OCT are summarized in Table 2.

**Fig. 1:**
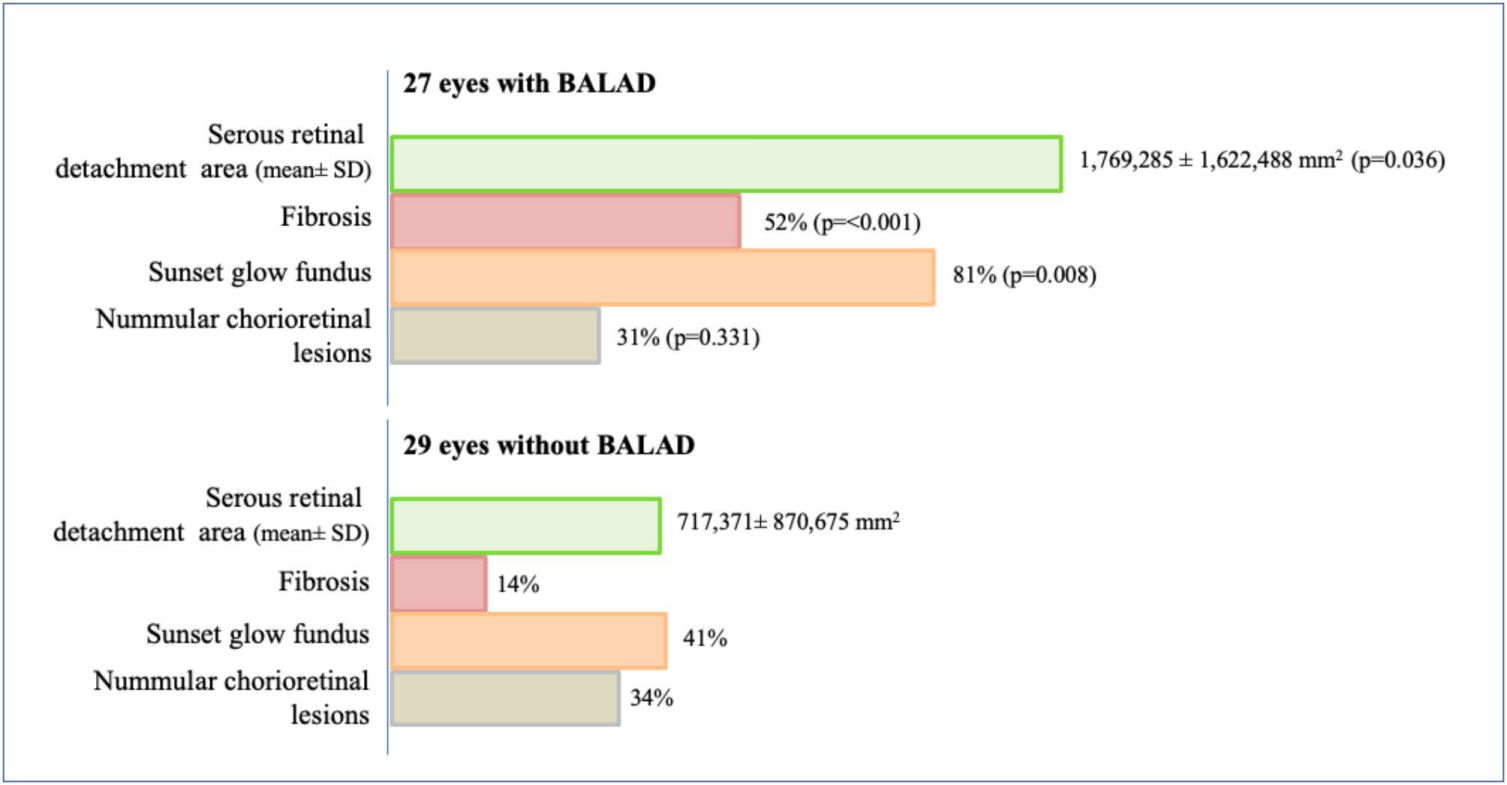
Association of presence of bacillary layer detachment (BALAD) at baseline with clinical parameters and long-term complications of 33 patients with Vogt-Koyanagi-Harada disease followed for 12 months since acute disease onset. *P* value represents the significance between eyes with BALAD and eyes without BALAD

**Fig. 2:**
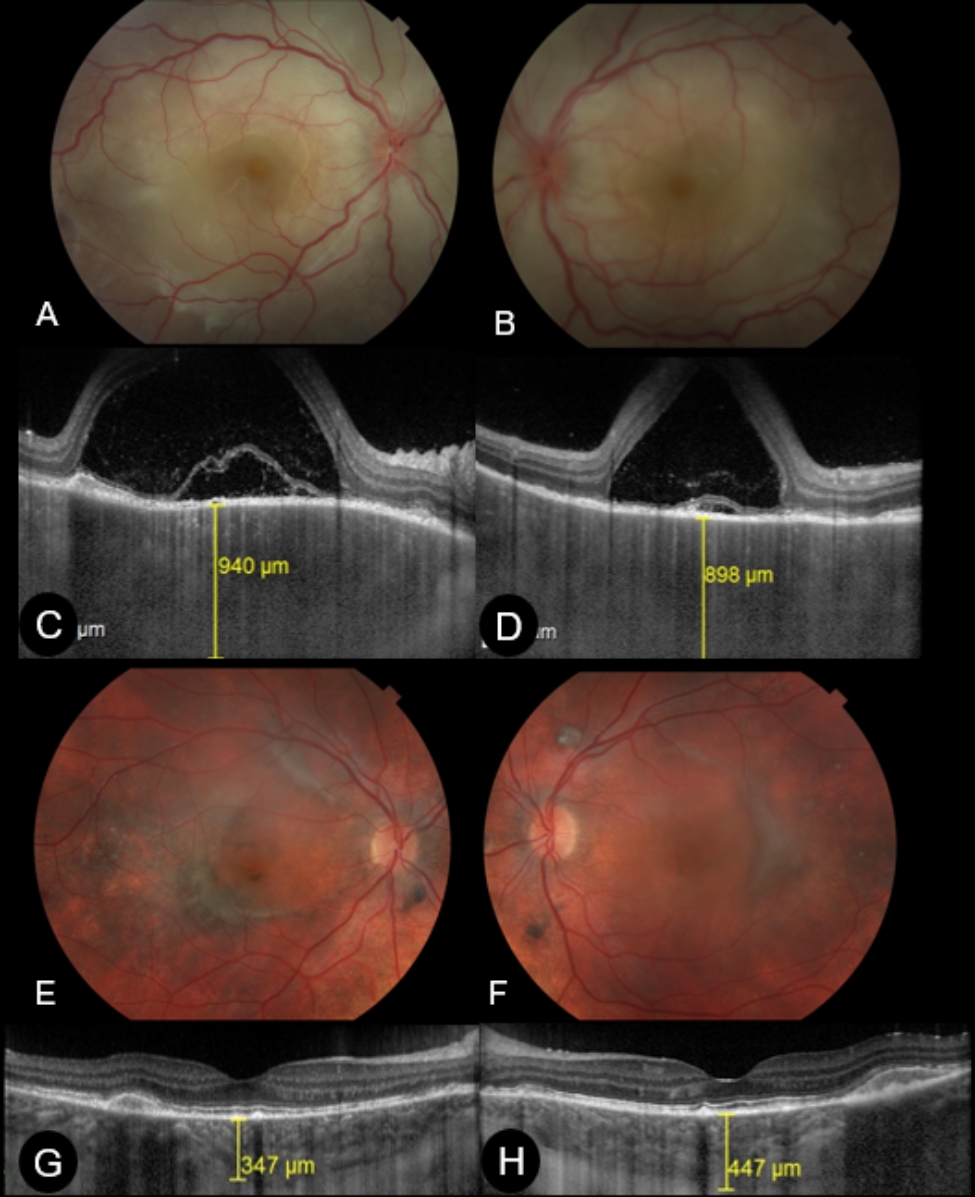
Multimodal imaging of a 26-year-old male patient diagnosed with Vogt-Koyanagi-Harada disease 32 days after disease onset. Initial visual acuity was counting fingers in both eyes. Treatment consisted of 3-day methylprednisolone pulse therapy followed by 50 mg of oral prednisone (1 mg/kg/day). At M1, best-corrected visual acuity (BCVA) was 0.3 logMAR in the right eye and 0.2 logMAR in the left eye. At M12, BCVA was 0 logMAR at both eyes and was taking 2.5 mg of prednisone and 150 mg of azathioprine. Initial retinal imaging revealed bilateral, extensive serous detachment and optic disc hyperemia and edema in both eyes (**A** and **B**). Enhanced Depth Imaging Optical Coherence Tomography (EDI-OCT), horizontal single scan through the fovea at initial presentation, there was an increased choroidal thickness and serous retinal detachment (SRD) with bacillary layer detachment (BALAD) (**C**, right eye; **D**, left eye). Retinal imaging at 12 months of follow-up demonstrated fundus depigmentation (sunset glow fundus), areas of atrophy, subretinal fibrosis and pigment disruption in both eyes (**E** and **F**). Enhanced depth imaging optical coherence tomography, horizontal single scan over the fovea, showed thickened choroid and subretinal fibrosis in the right eye (**G**) and in the left eye (**H**)

**Fig. 3:**
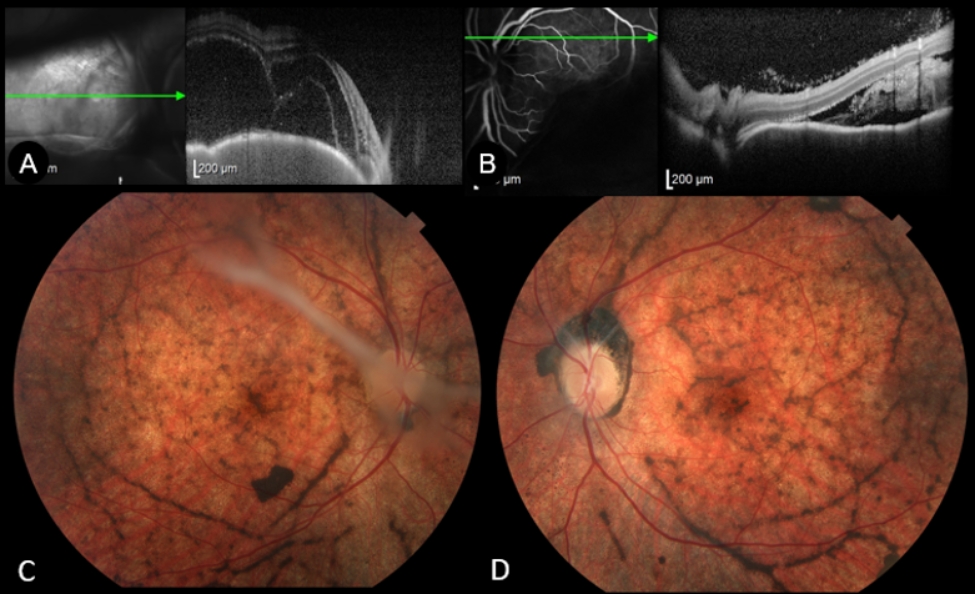
Spectral-domain Optical Coherence Tomography (SD-OCT), horizontal single scan, at initial presentation, with higher magnification, showing bacillary layer detachment (BALAD) in the right eye (**A**), and undefined subretinal hyperreflective material in the left eye (**B**) of 39-year-old female patient, diagnosed with Vogt-Koyanagi-Harada disease 27 days after symptoms onset. She underwent a 3-day methylprednisolone pulse therapy, followed by 50 mg prednisone (1 mg/kg/day) and 100 mg azathioprine (2 mg/kg/day). At the 12-month follow-up, retinal imaging revealed fundus depigmentation (sunset glow fundus) and subretinal fibrosis in both eyes (**C** and **D**)

Eyes with BALAD at baseline (*n* = 27; 48.2%) were associated with a higher number of eyes with altered ellipsoid zone (*P* = 0.01) and largest area of ​​serous detachment at baseline (*P* = 0.04) (Fig. 1). In addition, the presence of BALAD at M0 was associated with retinal structural changes at the first-year follow-up when compared to eyes without BALAD at M0, i.e., SRFib (51.9% vs. 13.8%; *P* < 0.001) and SGF (81.5% vs. 41.4%; *P* = 0.008). In the multivariate logistic regression analysis, the presence of BALAD at presentation (OR, 12.04; 95% CI 1.75 to 82.6; *P* = 0.011) and the interval to treatment initiation (32 days vs. 17 days; OR, 1.17; 95% CI 1.05 to 1.3; *P* = 0.004) were risk factors for SRFib at M12 (Table 3). Anterior uveitis flares (OR, 30.94; 95% CI 1.64 to 584.64; *P* = 0.022) and the presence of BALAD at presentation (OR, 14.78; 95% CI 2.74 to 79.6; *P* = 0.002) were risk factors for SGF at M12 (Table 3). Figures 2 and 3 demonstrated the multimodal imaging of two patients with persistent BALAD until M1 who had SRFib and SGF at M12.

**Table 3:** Subretinal fibrosis, sunset glow fundus, and nummular chorioretinal lesions within the first year and their associations with clinical and imaging parameters in 66 eyes of 33 patients with Vogt-Koyanagi-Harada disease followed for 12 months from acute onset

Subretinal fibrosis									
Parameters		Yes (*n* = 20)	No (*n* = 46)		Univariate analysis *P*	Multivariate analysis Odd ratio *P* CI 95%			
Interval to start treatment, days, mean ± SD		34 ± 17.13	20.3 ± 13.73		**0.025**	1.17	**0.004**	1.05–1.3	
median (interval)		32.5 (8–67)	17 (3–57)						
FA total score at M0, mean ± SD		8.47 ± 2.01	5.83 ± 2.90		**0.037**	4.06	**0.004**	1.57–10.5	
median (interval)		9 (5–13)	5.5 (0–9)						
SRD at M3, eyes (%)	Yes	5 (83.3)	1 (16.7)		**0.03**	4.53	0.337	0.21–98.89	
	No	15 (25.0)	45 (75.0)						
BALAD at M0, eyes (%)	Yes	14 (51.9)	13 (48.1)		**< 0.001**	12.04	**0.011**	1.75–82.6	
	No	4 (13.8)	25 (86.2)						
**Sunset glow fundus**									
Parameters		Yes (*n* = 38)		No (*n* = 28)	Univariate analysis *P*	Multivariate analysis Odd ratio *P* CI 95%			
Interval to start treatment, days, mean ± SD		30.29 ± 16.95		16.54 ± 10.54	**0.023**	1.08	0.069	0.99–1.17	
median (interval)		27 (8–67)		13 (3–44)					
AU recurrence in the first year, eyes (%)	Yes	21 (80.8)		5 (19.2)	**0.032**	30.94	**0.022**	1.64–584	
	No	17 (42.5)		23 (57.5)					
SRD area at M0, µm, mean ± SD		1,641,144 ± 1,564,898		517,212 ± 572,009	**0.004**	1	0.147	1	
median (interval)		1,178,291 (10368-5999296)		299,206 (8713-2448347)					
BALAD at M0, eyes (%)	Yes	22 (81.5)		5 (18.5)	**0.008**	14.78	**0.002**	2.74–79.6	
	No	12 (41.4)		17 (58.6)					
**Nummular chorioretinal lesions**									
Parameters		Yes (*n* = 26)		No (*n* = 40)	Univariate analysis *P*	Multivariate analysis Odd ratio *P* CI 95%			
Interval to start treatment, days, mean ± SD		33.19 ± 17.15		18.78 ± 12.45	**0.018**				
median (interval)		29.5 (8–67)		16 (3–51)					
FA total score at M0, mean ± SD		8.26 ± 2.03		5.68 ± 2.95	**0.042**				
median (interval)		9 (5–13)		5.5 (0–9)					

AU: anterior uveitis; BALAD: bacillary layer detachment; FA: fluorescein angiography; M: month; SRD: serous retinal detachment

The occurrence of late NCL was not associated with the presence of BALAD at presentation (Table 3).

Additional clinical analyses were performed to investigate associations between relevant systemic treatment variables and the main outcomes at 12 months. Specifically, we evaluated the timing of IMT (early vs. late), corticosteroid monotherapy versus combination therapy. None of these parameters showed statistically significant associations with SRFib, sunset glow fundus, or NCL at month 12 (Supplementary table).

## Discussion

In our cohort, we found early BALAD to be a common and strong predictor of long-term complications of VKHD. In addition, we reiterated that the interval to treatment from disease onset and the anterior uveitis recurrences were also independent predictors of poor outcomes, as SRFib and SGF.

“Bacillary layer detachment”, or BALAD, was first described by Mehta et al. in 2021. It was described as an OCT finding of photoreceptor splitting or shearing at the level of the myoid zone (between the external limiting membrane and the photoreceptor inner segment ellipsoid) adjacent to chorioretinal inflammation in a patient with macular active toxoplasmosis and pachychoroid disease spectrum. This OCT finding represented an inherent weakness in photoreceptor structure at this level during episodes of intense chorioretinal inflammation, based on similarity of their findings to a postmortem histologic artifact [9]. However, several Japanese researchers have previously studied, in acute VKHD, the OCT findings representing the same understanding of BALAD. For example, Ishihara et al. in 2009, with the advent of enhanced SD-OCT, brought the understanding of the outer retina morphological changes in the acute phase of VKHD as the outer segment becoming detached from the photoreceptor layer (“intraretinal split”) and the membranous structures composed of some retinal tissue besides amorphous fibrin [17]. 

Among the 66 eyes from 33 patients with VKHD included in our study, BALAD was detected in 50% at disease onset, whereas SRD was observed in up to 96%, consistent with VKHD being the most common non-infectious uveitis associated with SRD [18]. Previous studies have reported variable BALAD prevalence: Agarwal et al. (2020), using swept-source OCT, identified BALAD in 95% of 124 eyes [11], while Atas et al. (2021) and Kwak et al. (2023) reported rates of 57% and 59%, respectively [19, 20]. These discrepancies may reflect differences in BALAD definition, image interpretation, and OCT technology. Concerning BALAD resolution, it resolved earlier than SRD, in line with the findings of Agarwal et al., who reported earlier resolution for BALAD (3.4 ± 1.3 days) compared to SRD (5.9 ± 2.6 days). However, since our assessments were not conducted daily, but at baseline, month 1 (M1), and every three months thereafter, we could observe that at M1, SRD persisted in 42% of eyes, while BALAD was present in only 6%. Differences in resolution dynamics may be influenced by disease severity and treatment strategies. All patients were treated with 3-day methylprednisolone pulse therapy, followed by oral prednisone (1 mg/kg/day), and two-thirds had additional azathioprine or mycophenolate. In contrast, Agarwal et al. administered up to five day-pulse therapy in addition to early antimetabolites in all cases. In our cohort, despite variability in the timing of immunosuppressive initiation, early non-steroidal immunosuppression was not associated with one-year outcomes.

Previous early disease severity biomarker studies have implicated BALAD. Atas et al. observed that BALAD was more prevalent in patients with baseline SRD height exceeding 500 μm, while Kwak et al. observed an association between BALAD and recurrence features [19, 20]. Our data also demonstrated an association of the presence of BALAD at baseline with a higher SRD area, suggesting a more intense inflammatory response [19]. Furthermore, in our cohort, with a longer follow-up and systematic evaluation of inflammation, retinal structural changes and functional damage, the presence of BALAD at baseline was associated with markedly elevated rates of both SRFib and SGF at M12, pointing to a more severe course.

Even though a direct association of the presence of BALAD at baseline and visual outcomes were not observed in our study, the presence of BALAD at baseline was associated with a more disrupted ellipsoid zone. A disrupted ellipsoid zone at the third month was associated with subnormal ffERG parameters. Visual function abnormalities found more frequently in eyes with BALAD could be justified by the understanding that myoid zone split compromises the photoreceptor architecture, separating the inner and outer segments of the nucleus (IS/OS),^10^ and, that the thickness of myoid zone reflects the level of the metabolic activity of the photoreceptors [21]. While Kwak et al. found worse baseline visual acuity in eyes with BALAD, there was no difference in visual acuity between eyes with or without BALAD at six months [20]. In our study, we found an association of BALAD at baseline and structural outcomes at M12. Recently, Souto et al. described, in non-acute VKHD patients, an association of disrupted EZ with worse N1 and P1 amplitudes on multifocal ERG and with decreased mean deviation value on standard automated perimetry [22]. Furthermore, da Silva et al. found an association of fundus severity findings with retinal function measured by ffERG [14]. Therefore, the less intact EZ and IZ at M1, their longer recovery time, together with abnormal ffERG at M12, observed in our study, strengthened our hypothesis that the presence of BALAD at baseline is a biomarker of severity and worse prognosis, despite the good visual acuity.

Concerning the long-term complications outcomes, roughly one-third of the eyes developed SRFib within the first year of follow-up, within the literature range (8 to 40%)^1^. In our multivariate analysis, the presence of BALAD at baseline increased the risk of SRFib formation by twelvefold– a generally uncommon, although vision threatening, complication [15, 23, 24]. The hyperacute and aggressive choroidal inflammatory process in some patients with VKHD may be responsible for both the presence of BALAD and the increased risk of fibrosis progression, either as a result of more intense subretinal exudation causing vector shear forces on the photoreceptor, and the interaction between exudation products and the RPE [25, 26]. Concerning SGF, it was observed in slightly over 50% of the patients through the end of the first year. Suboptimal early treatment and chronicity of the inflammation are known to be associated with SGF development [27, 28]. We should note that at M12, 22 patients (66.7%) remained on prednisone (median 10 mg/day) and 14 of these patients had SGF suggesting the need of maintenance of treatment due to persistent inflammation. Some authors have shown that early dual immunosuppressive therapy in addition to corticosteroids, reduced to zero progression to SGF [29, 30]. In our study, we did not find an association between different groups of treatment and SGF, but we did find that anterior uveitis recurrences were associated with the development of SGF. Furthermore, our study brought a novel understanding of the presence of early BALAD as an independent risk factor to SGF.

Bacillary layer detachment is an increasingly recognized OCT biomarker of numerous heterogeneous chorioretinal diseases, including inflammatory and non-inflammatory disorders [10, 31, 32]. The imaging similarities between these entities suggest overlapping disease processes. Ramtohul et al. postulated that the main pathophysiological mechanism in BALAD genesis is comparable to exudative retinal detachment and involves breakdown of the RPE component of the outer blood-retina barrier with ELM integrity. These authors also pointed out to the choroidal and choriocapillaris flow impairments with increased choroidal thickness in these disorders [10]. Mehta et al. exposed two main factors required to form a BALAD, i.e., the potential space associated within the anatomic plane of weakness of the photoreceptor IS myoid bounded by the ELM and EZ and a hydrostatic force from the choroid strong enough to split the photoreceptors [9]. Among non-inflammatory disorders, we could list age-related macular degeneration, blunt ocular trauma, neoplastic and paraneoplastic retinal disorders; among inflammatory disorders, we could mention besides toxoplasmosis and VKHD, tubercular choroiditis and acute posterior multifocal placoid pigment epitheliopathy. BALAD often resolves spontaneously or with appropriate medical management [32]. 

We should mention the limitations of the present study. First, the small sample size, retrospective design and imaging interval limitations (monthly and not daily) could impact on multivariate models that, in spite of high ORs, there was a wide CI suggesting instability and also increasing Type I error risk. Second, at baseline ten out of 66 eyes were not included in the analysis due to image quality, risking selection bias and may impact on generalizability. And lastly, even though treatment heterogeneity could be a potential confounding factor, we should emphasize that the initial treatment for acute phase concerning corticosteroid was uniform for all participants (pulse therapy, high-dose oral prednisone and prednisone taper protocol). Treatment differed concerning association of azathioprine (corticosteroid only, corticosteroid and early azathioprine and corticosteroid and late azathioprine), but, bivariate analysis concerning treatment groups and presence of BALAD did not show significant impact on the results. Therefore, the present study results should be further validated in future larger cohorts.

In summary, this study clearly demonstrated the relevance and implication of BALAD at baseline in VKHD throughout a 12-month follow-up following acute onset of disease.This is the first retrospective cohort-based study involving a reasonable number of cases that explores the associations of BALAD as a biomarker of VKHD severity in a Brazilian cohort. In conclusion, BALAD was present, at baseline, in half of VKHD patients, and this finding was strongly correlated with a more intense early inflammation and the long-term development of both SRFib and SGF.

## Electronic supplementary material

Below is the link to the electronic supplementary material.

Supplementary Material 1

## Data Availability

No datasets were generated or analysed during the current study.

## Abbreviations

ART: Automatic real-time
BALAD: Bacillary layer detachment
EDI-OCT: Optical coherence tomography and enhanced depth imaging
EZ: Ellipsoid zone
FA: Fluorescein angiography
ffERG: Full-field electroretinogram
GEE: Generalized estimation equations
HCFMUSP: Hospital das Clínicas da Faculdade de Medicina da Universidade de Sao Paulo
HRA: Heidelberg retinal angiography
ICGA: Indocyanine green angiography
IZ: Interdigitation zone
M: Months
NCL: Nummular chorioretinal lesions
OCT: Optical coherence tomography
OR: Odds ratio
RPE: Retinal pigment epithelium
SD-OCT: Spectral domain optical coherence tomography
SGF: Sunset glow fundus
SRD: Serous retinal detachment
SRD: Serous retinal detachments
SRFib: Subretinal fibrosis
SRFld: Subretinal fluid
VKHD: Vogt-Koyanagi-Harada disease
