# Supplementary material for: Bacillary layer detachment in acute Vogt-Koyanagi-Harada disease: an early predictor of long-term complications in a Brazilian cohort
Source: Int J Retina Vitreous. 2025 Apr 22;11:50. doi: 10.1186/s40942-025-00673-6 (PMC12013148; doi:10.1186/s40942-025-00673-6)
Supplement: Supplementary file 1 — Supplementary Material 1 [file 40942_2025_673_MOESM1_ESM.docx]

**Supplementary table –** Structural and functional outcomes at one year and their association with treatment groups in 66 eyes of patients with Vogt-Koyanagi-Harada disease followed for 12 months from acute onset

| **First-year outcomes** | **SRFib** | *P* | **SGF** | *P* | **NCL** | *P* | **Subnormal**  **ffERG^a^** | *P* |
| --- | --- | --- | --- | --- | --- | --- | --- | --- |
|  | (n=20 eyes) |  | (n=38 eyes) |  | (n=26 eyes) |  | (n=47 eyes) |  |
| Treatment, patients (%) |  | 0.773 |  | 0.339 |  | 0.361 |  | 0.672 |
| Corticosteroid monotherapy | 7 (35.0) |  | 8 (40.0) |  | 5 (25.0) |  | 16 (80.0) |  |
| Corticosteroid + early immunosuppressive therapy (≤1mo) | 5 (22.7) |  | 13 (59.1) |  | 8 (36.4) |  | 12 (66.7) |  |
| Corticosteroid + late immunosuppressive therapy (>1mo) | 8 (33.3) |  | 17 (70.8) |  | 13 (54.2) |  | 19 (79.2) |  |

ffERG: full-field electroretinogram; NCL: nummular chorioretinal lesions; SGF: sunset glow fundus; SRFib: subretinal fibrosis.

^a^Subnormal ffERG was defined as having at least one parameter below the 5^th^ percentile of healthy age and gender-matched controls.
